# Synthesis of silver nanoparticles embedded into melamine polyaminal networks as antibacterial and anticancer active agents

**DOI:** 10.1038/s41598-024-70606-0

**Published:** 2024-08-28

**Authors:** Maha M. Alotaibi, Bodoor Almalki, Nada Tashkandi, Fatemah Basingab, Samaa Abdullah, Nazeeha S. Alkayal

**Affiliations:** 1 Chemistry Department, Faculty of Science, King Abdulaziz University, P.O. Box 80203, 21589 Jeddah, Saudi Arabia; 2 Department of Biological Sciences, Faculty of Science, King Abdulaziz University, P.O Box 80200, 21589 Jeddah, Saudi Arabia; 3 Immunology Unit, King Fahad Medical Research Centre, King Abdulaziz University, P.O Box 80200, 22252 Jeddah, Saudi Arabia; 4 College of Pharmacy, Amman Arab University, Amman, 11953 Jordan; 5 Creativity, Innovation and Entrepreneurship Center, Amman Arab University, Amman, 11953 Jordan

**Keywords:** Porous organic polymer, Melamine-based polyaminal, Silver nanoparticles, Antibacterial, Anticancer, Biochemistry, Biological techniques, Chemistry

## Abstract

Silver nanoparticles were successfully incorporated into a melamine-based polymer, resulting in the synthesis of (Ag NPs@Bipy-PAN) through a reverse double solvent approach. The synthesised Ag NPs@Bipy-PAN polymer underwent extensive characterisation through Powder X-ray Diffraction (PXRD), Scanning Electron Microscopy (SEM), Transmission Electron Microscopy and Energy Dispersive X-ray (EDX) and Thermal Gravimetric Analysis. PXRD analysis confirmed the successful encapsulation of Ag nanoparticles and provided insights into the amorphous nature of the polymer following encapsulation. SEM and EDX analyses further corroborated the presence and distribution of Ag nanoparticles on the polymer surface. The biological efficacy of the Ag NPs@Bipy-PAN polymer was evaluated through antibacterial, anti-breast cancer, and biocompatibility assays. The results demonstrated notable antibacterial and anticancer activities, with significant efficacy against bacterial strains and breast cancer cells. Biocompatibility assessments indicated acceptable compatibility, particularly at a concentration of 2.5 mg/mL, compared to untreated control cells. These findings suggest that Ag NPs@Bipy-PAN has considerable potential as a candidate for cancer-targeted and antimicrobial drug delivery systems. The incorporation of silver nanoparticles into the melamine-based polymer enhances the safety profile of these systems in in vivo conditions, making them a viable option for advanced therapeutic applications.

## Introduction

One of the most crucial modern day health concerns is the antibacterial resistance, as pathogens continually adapt to resist traditional antibiotic remedies, resulting in diminished treatment effectiveness. However, despite the imperative need to address this crisis, the discovery of new antimicrobial compounds has notably declined. Conversely, the prevalence of pathogen resistance to widely prescribed antibiotics has significantly increased. This alarming trend underscores the critical need for the exploration and development of novel therapeutic and preventive antimicrobial interventions^1,2^.

In response to this urgent challenge, the exploration of novel antimicrobial agents such as melamine-based polyaminal polymers (PANs) has gained considerable attention in recent years^3^. PANs possess distinctive properties, including a high nitrogen content and the presence of amino groups, which impart them with antimicrobial activity^4^. Melamine, a compound consisting of cyanamide and 1,3,5 triazine, is recognised for its nitrogen-rich structure, allowing it to impart excellent sorption capabilities and chemical stability when used in the creation of porous polymers^4^. These polymers are synthesised through a one-pot polycondensation reactions and microwave methods, resulting in various interconnected networks such as azolinked polymer^5^, polyamide^5,6^, polyimide^5,6^, and polyaminal networks^7^. PANs are particularly of a significant importance due to their nitrogen-rich composition from triazine rings and aminal linkages^8^, along with features like abundant micropores^9^, a large surface area^9^, and simplified synthesis via one-pot catalyst-free polycondensation of aldehyde monomers combined with varieties of amines^10^.

Empirical investigations have demonstrated the broad-spectrum inhibitory effects of PANs against diverse bacterial strains, encompassing both Gram-positive and Gram-negative species. The underlying mechanisms of PANs' antibacterial action primarily involve interactions with bacterial cell membranes and intracellular components^11,12^. Positively charged amino groups within PANs facilitate electrostatic interactions with negatively charged bacterial cell surfaces, leading to membrane disruption and subsequent cellular lysis^13^. Furthermore, the nitrogen-rich composition of PANs may disrupt bacterial metabolic processes, contributing to their antimicrobial efficacy^14^. These multifaceted mechanisms underscore the potential of PANs to serve as promising candidates for combating antibacterial resistance.

Several subclasses of polyaminal polymers (PANs) have emerged, and each with their own distinctive characteristics and potential applications or antibacterial applications due to their ability to facilitate interactions with bacterial membranes and intracellular components^15^. Polymers with intrinsic microporosity (PIMs) exhibit inherent microporosity owing to their rigid and contorted structures^16^, which may contribute to enhanced antibacterial activity through mechanisms such as membrane disruption and inhibition of bacterial metabolic processes^17–19^. Covalent organic frameworks (COFs) represent another subclass with potential antibacterial properties, characterized by crystalline porous materials formed through the self-assembly of organic building blocks, offering tuneable porosity and surface functionality advantageous in combating bacterial infections^20,21^. Covalent triazine-based frameworks (CTFs), featuring triazine units linked by covalent bonds, provide high surface areas and thermal stability^22^, which may contribute to their efficacy in antibacterial applications by facilitating interactions with bacterial surfaces and promoting antimicrobial activity. Hyper-cross-linked polymers (HCPs)^23^ and porous aromatic frameworks (PAFs) also hold promise for antibacterial applications, offering high porosity and surface areas conducive to interactions with bacterial membranes and intracellular components, thus potentially enhancing their antibacterial efficacy^24^.

On the other hand, silver nanoparticles (AgNPs) are well-known for their antimicrobial properties, capitalize on their elevated surface area to volume ratio to outstrip bulk silver in antimicrobial efficacy^25^. Silver nanoparticles exhibit antibacterial effects through multiple mechanisms. Firstly, they release silver ions continuously, which adhere to bacterial cell walls and cytoplasmic membranes, enhancing permeability and disrupting the bacterial envelope^26^. Within cells, silver ions deactivate respiratory enzymes, generate reactive oxygen species, and interfere with DNA replication and protein synthesis. Additionally, silver nanoparticles themselves can kill bacteria by accumulating in cell wall pits, causing membrane denaturation, and disrupting organelles, leading to cell lysis. They can also penetrate bacterial cell walls and alter membrane structure, affecting signal transduction and promoting cell apoptosis^26,27^. The suspension of silver nanoparticles in exposure media impacts their antibacterial efficacy, with smaller nanoparticles and certain shapes releasing silver ions more efficiently^26,28^. Gram-negative bacteria possess thinner cells walls and are thus highly susceptible to silver nanoparticles. However, biofilms can hinder the effectiveness of silver nanoparticles by impeding their transport and reducing their diffusion coefficients, thereby allowing bacteria within the biofilm to remain tolerant to silver nanoparticle exposure^29^.

Functioning through multiple mechanisms such as membrane disruption, interference with cellular processes, and generation of reactive oxygen species, AgNPs have been effectively utilized in textiles, medical devices, and coatings as antimicrobial agents^30–32^. The incorporation of PANs and AgNPs may synergistically improve their antimicrobial properties^33^. Specifically, the positively charged amino groups inherent in PANs can facilitate the binding and subsequent release of AgNPs, fostering enhanced interaction with microorganisms. In this study, we demonstrate the antibacterial potential of one-pot polycondensation bipyridine-based Polyaminal encapsulated with Ag nanoparticles (Ag NPs@Bipy-PAN).

## Material and methods

### Materials

The chemicals used in this study did not undergo any further purification. [2,2′-Bipyridine]-5,5′-dicarbaldehyde was purchased from Shanghai Sunchem Inc., Shanghai, China. Melamine (97.5%), silver acetate (CH_3_COOAg 98.5%), and dimethyl sulfoxide (DMSO 99%) were supplied by BDH Laboratory Reagents, England, UK. Tetrahydrofuran (THF ≥ 99.5%), acetone (99.5%), dichloromethane (DCM ≥ 99.9%), and Pd(NO_3_)_2_ (99%) were purchased from Fisher Scientific, Chicago, USA. NaOH (98%), NaBH_4_ (99%), and (CH_3_.COO)_2_ Cd.2H_2_O (99%) were supplied by BDH Chemicals, England, UK. NiSO_4_.6H_2_O (98%) and (CH_3_COO)_2_ Cu.H_2_O (≥ 99.8%) were obtained from BDH Laboratory Supplies, England, UK. Ba(NO_3_)_2_ (99%) was provided by Ward's Natural Science, Rochester, NY, USA, and finally, HCl (35%) was supplied by LOBA Chemie, Mumbai, India.

### Synthesis of bipyridine-based polyaminal-linked porous organic polymer (Bipy-*PAN*)

Melamine-based porous polyaminal was prepared according to a previous study^34^. The synthesis involved utilizing a dry three-necked flask equipped with a magnetic stirrer, and the condenser was degassed through an evacuation-argon-backfill cycle. Initially, a vacuum was applied to evacuate the flask. Melamine (0.5 g, 3.96 mmol) and [2,2′-Bipyridine]-5,5′-dicarbaldehyde (0.7 g, 5.94 mmol) were dissolved in 30 mL of DMSO. The resulting mixture was then subjected to heating at 175 °C for 3d, with continuous stirring under an argon flow. The precipitate was obtained by filtration and subjected to washing with dimethylformamide (3 times), dichloromethane (3 times), and acetone (3 times). Subsequently, the white solid product was dried under vacuum at 70 °C for 2 h, resulting in a yield of 72%.

### Synthesis of the Ag NPs@Bipy-*PAN*

To encapsulate Ag NPs by Bipy-PAN, 170 mg of Bipy-PAN powder was suspended in 40 mL of deionized water as a hydrophilic solvent, and the mixture was sonicated for 1 h until it became homogeneous. After stirring for 30 min, a solution of ${\text{CH}}_{3}\text{COOAg}$(0.02 mmol) dissolved in $\text{DCM}$ 0.04 mL) as the hydrophobic solvent was added dropwise for 10 min with constant vigorous stirring. The resulting solution was continuously stirred for 5 h. Then, the as-prepared mixture was reduced by adding a highly concentrated ${\text{NaBH}}_{4}$ aqueous solution (2.7 M, 1 mL). Finally, the mixture was filtered, washed with deionized water, and dried at 70 °C for 2 h to obtain the solid sample.

### Instrumentation

Using a Maxima XRD-7000X Powder X-ray Diffraction (PXRD) and Dispersive X-ray Spectroscopy (EDX) equipments (Shimadzu, Kyoto, Japan), Ag@BiPy-PAN was investigated. Using nickel-filtered Cu-Kb reduction, X-rays were produced at 40 kV and 100 mA during the process. Ten degrees per minute was the scan speed, and the scan range (2θ) was 5 to 70 degrees. Ag@BiPy-PAN was investigated using Thermal Gravimetric analysis (TGA) on a TG-DTA6300 (Shimadzu, Kyoto, Japan) with a heating rate of 10 °C min^-1^ at intervals of 25–500 ° C in an N2 environment. The structure and particle distribution of Ag@BiPy-PAN in powder form were investigated using Scanning Electron Microscopy (SEM) (FEI Inspect F50, FEI, Tokyo, Japan). The dry substance was examined at a voltage of 30 kV. Additionally, the Ag@BiPy-PAN's size and morphology were examined using Transmission Electron Microscopy (TEM) (JEM-F200, Jeol, Tokyo, Japan). Following negative staining (phosphotungstic acid, 2%) with one drop of the substance applied to a copper grid at the proper dilution, TEM images were obtained^35^.

### Antibacterial assay

Gram-negative *Escherichia coli* (*E. coli,* ATCC 25,922) and gram-positive *Staphylococcus aureus* (*S. aureus,* ATCC 25,923) were selected to examine the antibacterial effects of BiPy-PAN and Ag@BiPy-PAN using the agar well diffusion method. In this method, bacterial cells were sub-cultured in nutrient broth agar for 24 h before compound antibacterial testing to ensure bacterial cells were in the log phase. Mueller–Hinton agar (MH) media were prepared and inoculated by spreading bacterial inoculum over the whole agar surface. Then holes of 8 mm diameter were punched using a sterile Cork borer to add 100 µl of 0.25 mg, 0.5 mg, and 1 mg of the compound under investigation. Bacterial cells were incubated in a humified incubator under sterile conditions at 37 °C for 24 h in the presence, or absence, of the compounds. The inhibition zone around the hole was measured using a calibre^36^.

### The cell viability assay

Breast cancer cells (MCF-7) and immobilized human embryonic kidney cells (HEK) were maintained, and cultures following the American Type Culture Collection (ATCC) guidelines and purchased from ATCC, Manassas, Virginia. The cultured cell lines were grown under 37 °C, 80–90% confluence, and 5% CO_2._ For the cell viability, the MTT assay kit was procured from Invitrogen, USA. Cells were initially left for incubation at 37 °C for 24 h using a 96-well plate^37^. In each well, 2 × 10^3^ of each cell type was placed and kept inside the humified carbon dioxide incubator, maintained at 5% humidity. The treatment group of of BiPy-PAN and Ag@BiPy-PAN was added to the well at different concentrations and left for 48 h. After this, all the samples were centrifuged, and precisely 100 µl of obtained supernatant was replaced with that of DMSO and again left for incubation at 5% carbon dioxide and 37 °C for 4 h. The absorbance was recorded at 570 nm in a triplicate using a microplate reader^35,36^.

## Results and discussion

### Synthesis of the Ag NPs@Bipy-*PAN*

**Scheme 1:**
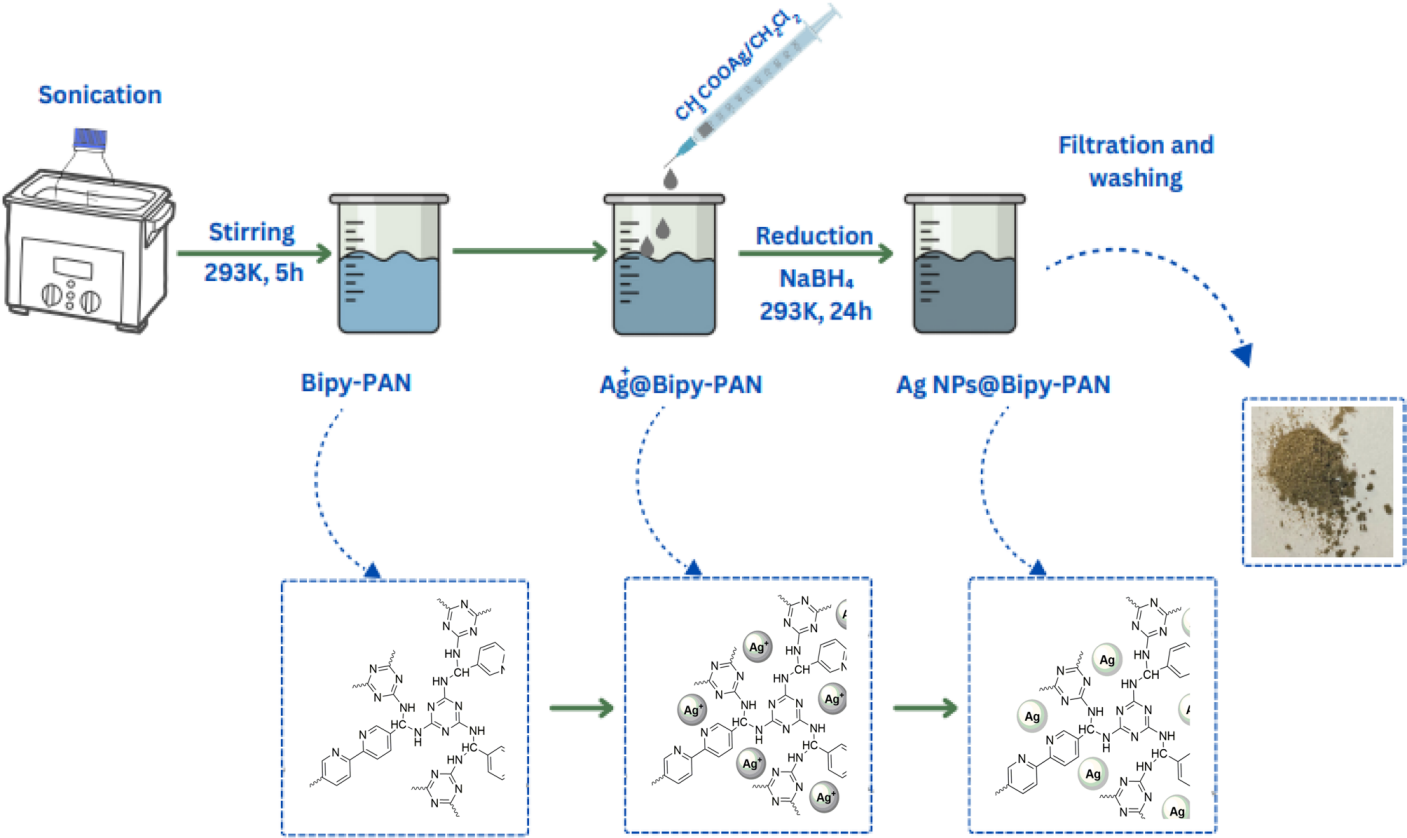
Preparation of Ag NPs@Bipy-PAN.

The Bipy-PAN polymer was utilized as an effective material for stabilizing metal ions by leveraging the binding sites provided by the nitrogen atoms in Bipyridine, triazine units, and aminal linkage. This indicates that the Bipy-PAN polymer has the potential to serve as an outstanding support for immobilizing metal nanoparticles. The reverse double solvent method (RDSM) was employed to investigate the immobilization of silver nanoparticles onto the polymer. The process involved dispersing the Bipy-PAN polymer in deionized water and adding a solution of CH_3_COOAg/CH_2_Cl_2_ (0.5 M). The mixture was then subjected to sonication, and after 5 h of stirring, NaBH_4_ was used as a reducing agent to convert the silver ions into atoms, resulting in the formation of a grey powder known as Ag NPs@Bipy-PAN (Scheme 1). Various characterization techniques, including PXRD, SEM, TEM, EDX, TGA, and N_2_ adsorption–desorption methods, were employed to confirm the successful formation of the Ag NPs@Bipy-PAN adsorption.

### PXRD analysis

**Fig. 1:**
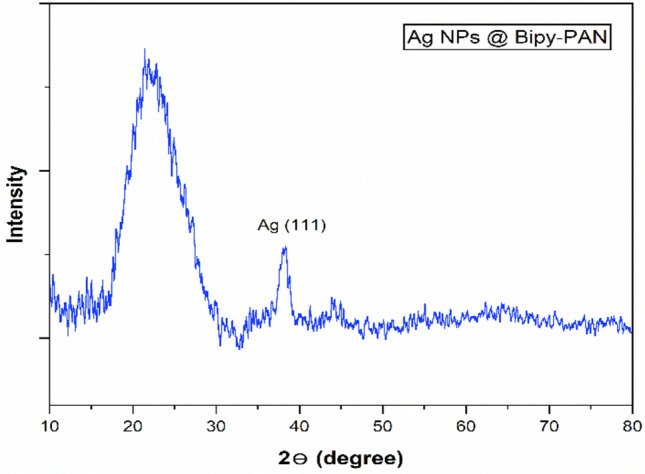
PXRD patterns of Ag NPs@Bipy-PAN.

The powder X-ray diffraction (PXRD) was used to determine the crystal type of polymer and the concentration ratio of silver nanoparticles within the polymer matrix. From (Fig. 1), it was observed that the Ag NPs@BiPy-PAN exhibited broad diffraction peaks at 2θ of 21°, which are characteristic of the amorphous structure of the BiPy-PAN polymer, as well as sharp diffraction peaks at 2θ of 38.2°, which correspond to the (111) diffraction planes of the face-centred cubic structure of silver nanoparticles (JCPDS No. 04–0783). These results suggest that the silver nanoparticles were able to distribute and encapsulate within the BiPy-PAN polymer without significantly altering its original structure^34^.

### Morphological analysis by SEM, TEM and EDX

**Fig. 2:**
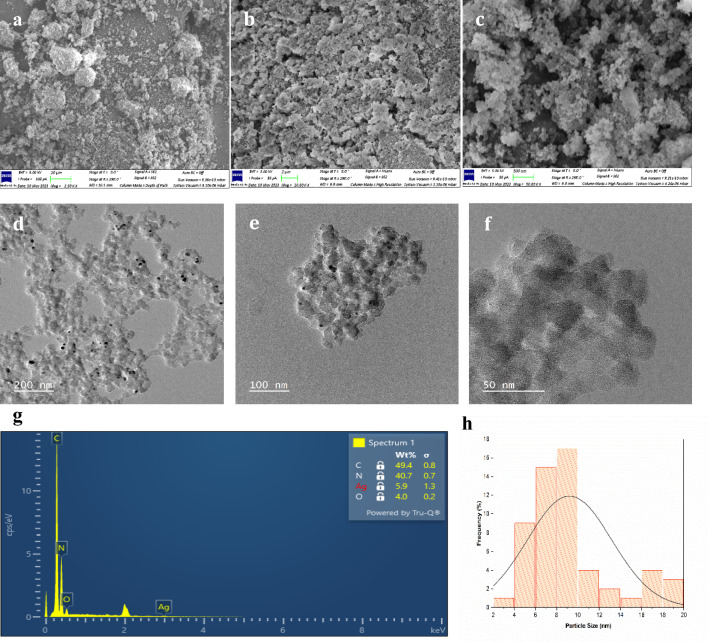
(**a**,**b**,**c**) SEM images with various magnifications of Ag NPs@Bipy-PAN. (**d**,**e**,**f**) TEM images with various magnifications of Ag NPs@Bipy-PAN. (**g**) Area EDX spectra of Ag NPs@Bipy-PAN. (**h**) Particle size distribution histogram.

The morphology, structure, and particle size distribution were thoroughly investigated using Scanning Electron Microscopy (SEM) and Transmission Electron Microscopy (TEM) analysis. The SEM image of Ag NPs@BiPy-PAN (Fig. 2a–c) showed a compact, irregular shape. However, the morphology of Ag@BiPy-PAN remained unchanged during AgNPs loading. The TEM images of Ag@BiPy-PAN displayed a spherical morphology of BiPy-PAN, with black dots representing Ag particles uniformly distributed on BiPy-PAN surface (Fig. 2d–f). The average particle size of Ag nanoparticles was 9.2 nm, likely due to the coordination between AgNPs and nitrogen active sites in BiPy-PAN (Fig. 2h). Energy dispersive X-ray (EDX) analysis indicated the presence of dispersed C, N, and Ag on the surface of Ag@BiPy-PAN, as shown in (Fig. 2e–g). Overall, the results demonstrated the successful anchoring of Ag nanoparticles onto BiPy-PAN without significant changes in its morphology^34^.

### TGA analysis

**Fig. 3:**
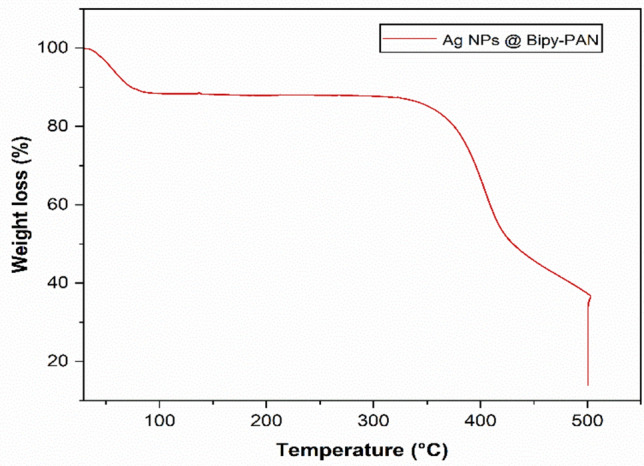
TGA analysis of Ag NPs@Bipy-PAN.

The thermogravimetric analysis (TGA) was used to assess the thermal stability of Ag NPs@BiPy-PAN. (Fig. 3) illustrates that the TGA curve of Ag NPs@BiPy-PAN showed an initial weight loss (about 10 wt%) in the temperature range of 35–80 °C, which can be attributed to the removal of absorbed water and solvent molecules. Furthermore, the material exhibits high thermal stability up to 360 °C, beyond which a significant weight loss (39.78 wt%) occurs in the temperature range of 360–460 °C, ascribed to the decomposition of the polymer network. The TGA analysis also indicates that Ag NPs@BiPy-PAN retains about 36.7% of its total weight at 500 °C. Overall, the results demonstrate that Ag NPs@BiPy-PAN exhibits outstanding thermal stability up to 360 °C^34^.

### Antibacterial effects

**Fig. 4:**
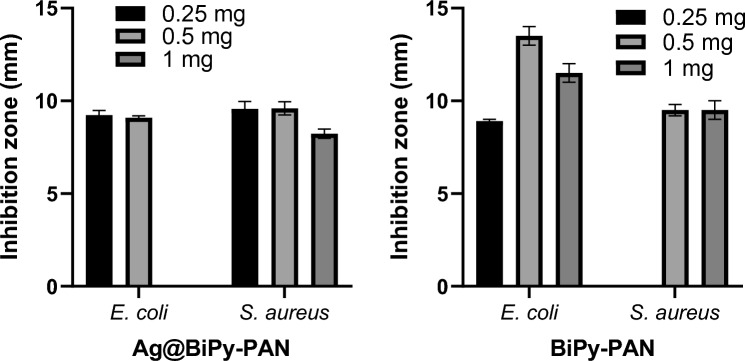
Antibacterial effects of Ag@BiPy-PAN and BiPy-PAN of on *E. coli* and *S. aureus*.

The effects of Ag@BiPy-PAN were examined against gram-negative *E. coli* and gram-positive *S. aureus* bacteria, in which selected bacteria were incubated either in the presence or in the absence of 100 µl of 0.25, 0.5 and 1 mg of the compounds. Table 1 represents the anti-bacterial effects of Ag@BiPy-PAN on *E. coli* and *S. aureus* by observing a clear inhibition zone around the compounds. Results in both Table 1 and Fig. 4 demonstrate that the Ag@BiPy-PAN compound created clear inhibition zones impacting the growth of both bacteria in comparison to the BiPy-PAN, especially at 0.25 mg, that could add to the safety profile. Ag@BiPy-PAN inhibited the growth of *E. coli* by creating an inhibition zone of 9 mm when using 0.25 mg and 0.5 mg of the compound but the clear inhibition zone was not detected despite that the bacterial growth was reduced by 14 mm around the compound with the addition of 1 mg (light zone). Moreover, Ag@BiPy-PAN is effective on *S. aureus* creating a 10 mm clear inhibition zone around the compound regardless of increasing concentrations. Structural differences between both types of bacteria underlie the different effects Ag@BiPy-PAN exhibited against them.

**Table 1:**
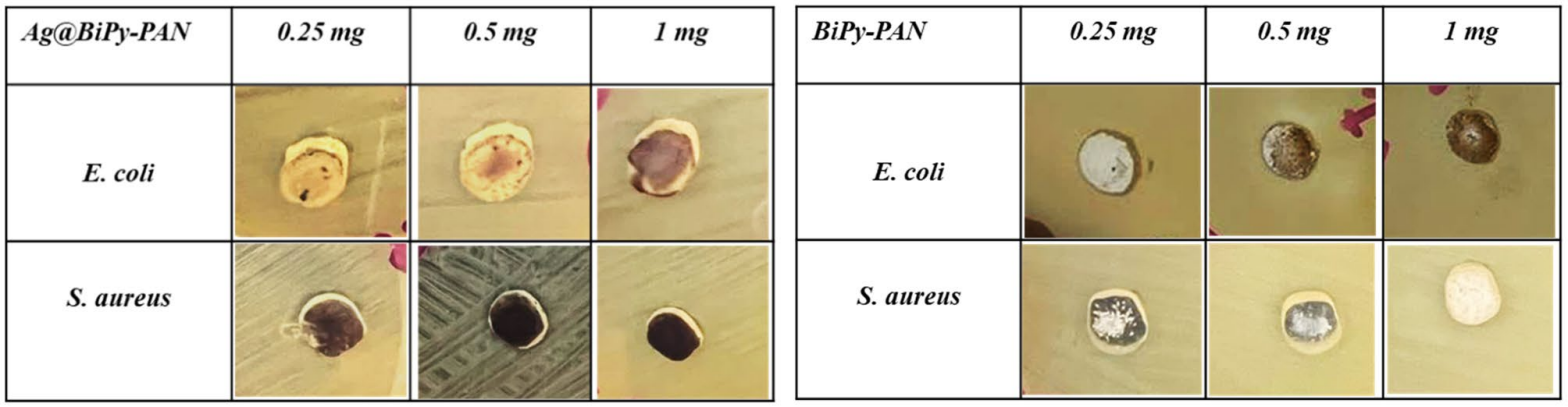
Antibacterial effects of Ag@BiPy-PAN and BiPy-PAN of on *E. coli* and *S. aureus*.

Due to the electrostatic interaction between the positively charged resin and negatively charged bacteria cell membrane, the Ag@BiPy-PAN resin could interfere with the balance and integrity of the bacterial cell wall exhibiting anti-microbial activity towards microorganisms. In addition, the free radicals, derived from the resin surface, could disturb the membrane lipids and structure of microorganism cells, leading to a breakdown of membrane functions ^36^.

### Anticancer and biocompatibility polymer effects

**Fig. 5:**
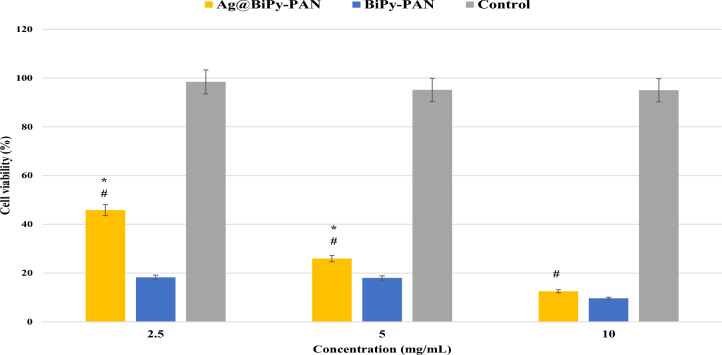
The MTT assay against MCF-7 of the Ag@BiPy-PAN and BiPy-PAN polymers. One-way ANOVA was to determine the significance between the Ag@BiPy-PAN and BiPy-PAN polymers (*), or between the Ag@BiPy-PAN and control (#), which the *P*-value of less than 0.05 was considered significant.

The cytotoxic effects of Ag@BiPy-PAN and BiPy-PAN are apparent in Fig. 5 against the breast cancer cells. The 50%-inhibitory concentrations (IC50) were 1.15 ± 0.11 mg/mL and 0.68 ± 0.21 mg/mL of Ag@BiPy-PAN and BiPy-PAN, respectively. As a result, the potency of the BiPy-PAN was higher than the Ag@BiPy-PAN. The BiPy-PAN in physiological conditions could be positively charged on the secondary amine functionality, which could enhance the BiPy-PAN cellular adherence to the bilayer phosphate group. Afterwards, the BiPy-PAN would imbalance the cellular membrane integrity causing cell death^37,39^. On the other hand, Ag@BiPy-PAN could follow the same mechanism, but the silver nanoparticles might interfere with the cellular adherence of the polymer, especially at concentrations lower than 2 mg/mL.

**Fig. 6:**
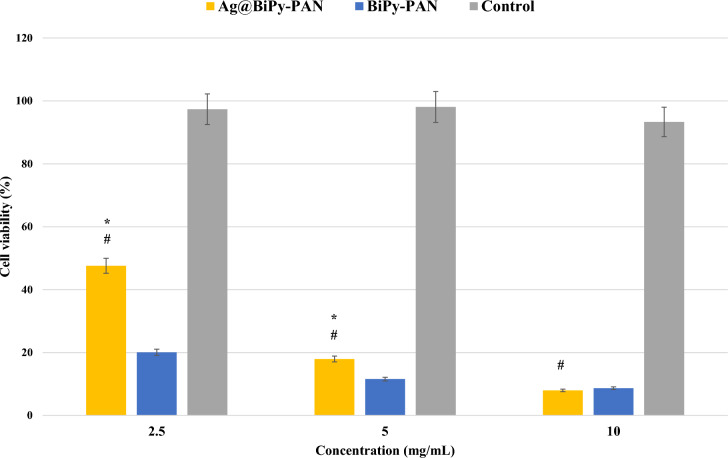
The MTT assay against HEK of the Ag@BiPy-PAN and BiPy-PAN polymers. One-way ANOVA was to determine the significance between the Ag@BiPy-PAN and BiPy-PAN polymers (*), or between the Ag@BiPy-PAN and control (#), which the P-value of less than 0.05 was considered significant.

Regarding the polymers’ effects on the immobilized kidney cells, the cytotoxic effects of Ag@BiPy-PAN and BiPy-PAN are apparent in Fig. 6 against the normal kidney cancer cells. The 50%-inhibitory concentrations (IC50) were 1.45 ± 0.11 mg/mL and 0.91 ± 0.21 mg/mL of Ag@BiPy-PAN and BiPy-PAN, respectively. As a result, the potency of the BiPy-PAN was higher than the Ag@BiPy-PAN. The mechanism of actions and behavior could be related to the above explanation. The mechanism of action could be confirmed to be un-specific to the cancer cells^37,39^. As a result, a cancer-targeted drug delivery systems of Ag@BiPy-PAN and BiPy-PAN could enhance their safety profile when used against cancer^38,39^.

## Conclusion

This study successfully synthesised and characterised silver nanoparticle stabilised by a bipyridine-based polymer (Ag NPs@Bipy-PAN). The Bipy-PAN polymer demonstrated significant potential as a support material for immobilising metal nanoparticles, utilising the binding sites provided by its nitrogen atoms. The reverse double solvent method (RDSM) facilitated the embedding of silver nanoparticles within the polymer matrix, as confirmed through various characterisation techniques including PXRD, SEM, TEM, EDX, TGA, and N2 adsorption–desorption methods.

PXRD analysis revealed that the silver nanoparticles were well-distributed within the amorphous structure of the Bipy-PAN polymer, maintaining its original integrity. SEM and TEM analyses showed consistent morphology and uniform distribution of Ag nanoparticles, with an average particle size of 9.2 nm. EDX confirmed the elemental composition, verifying the presence of Ag, C, and N on the polymer surface. TGA analysis demonstrated that Ag NPs@Bipy-PAN exhibited remarkable thermal stability up to 360 °C, indicating its suitability for high-temperature applications.

Functionally, Ag NPs@Bipy-PAN exhibited effective antibacterial activity against both gram-negative E. coli and gram-positive S. aureus, as evidenced by clear inhibition zones that increased with the compound concentration. This antibacterial effect is likely due to electrostatic interactions and free radicals derived from the resin surface, disrupting bacterial cell membranes. However, variations in nanoparticle size and polymer matrix can influence antibacterial efficacy.

The anticancer properties of Ag NPs@Bipy-PAN were assessed against breast cancer cells and normal kidney cells. The results indicated that while the BiPy-PAN polymer alone exhibited higher cytotoxicity, Ag NPs@Bipy-PAN also showed significant inhibitory effects. The data suggested a potential mechanism involving the interference of silver nanoparticles with cellular adherence, indicating a non-specific action on cancer cells.

Ag NPs@Bipy-PAN demonstrate considerable promise as a multifunctional material with applications in antibacterial treatments and cancer therapy. Future studies should focus on developing targeted drug delivery systems to enhance the specificity and safety profile of Ag NPs@Bipy-PAN and BiPy-PAN in clinical applications. The continued exploration of nanoparticle-polymer composites could lead to significant advancements in biomedical applications, reflecting a broader trend in the development of multifunctional nanomaterials for medical use.

## Data Availability

Data will be made available on request.

## References

[1] World Health Organisation (WHO), 2023, https://www.who.int/news-room/fact-sheets/detail/antimicrobial-resistance#:~:text=As%20a%20result%20of%20drug,through%20genetic%20changes%20in%20pathogens

[2] Antimicrobial Resistance Collaborators. Global burden of bacterial antimicrobial resistance, a systematic analysis. *The Lancet* **399**(10325), 629–655 (2019). 10.1016/S0140-6736(21)02724-0 PMC8841637 35065702

[3] Huang, K. S. *et al.* Recent advances in antimicrobial polymers: a mini-review. *Int J Mol Sci.* **17**(9), 1578 (2016). 27657043 10.3390/ijms17091578 PMC5037843

[4] Dorieh, A. *et al.* A review of recent progress in melamine-formaldehyde resin based nanocomposites as coating materials. *Progr. Org. Coat.* **165**, 106768 (2022). 10.1016/j.porgcoat.2022.106768

[5] Shao, L., Liu, M., Sang, Y. & Huang, J. One-pot synthesis of melamine-based porous polyamides for CO_2_ capture. *Microporous Mesoporous Mater.* **285**, 105–111 (2019). 10.1016/j.micromeso.2019.05.005

[6] Fawaz, J., & Mittal, V. Synthesis of polymer nanocomposites: review of various techniques. Synthesis techniques for polymer nanocomposites, 2014: 1–30.

[7] Fajal, S., Dutta, S., & Ghosh, S. K. Porous organic polymers (POPs) for environmental remediation. Materials Horizons, 2023: 4083. 10.1039/d3mh00672g 37575072

[8] He, D. *et al.* Synthesis and study of low-cost nitrogen-rich porous organic polyaminals for efficient adsorption of iodine and organic dye. *Chem. Eng. J.* **446**, 137119 (2022). 10.1016/j.cej.2022.137119

[9] Wang, B. *et al.* Nitrogen-rich porous biochar for highly efficient adsorption of perchlorate: Influencing factors and mechanism. *J. Environ. Chem. Eng.* **11**(3), 110293 (2023). 10.1016/j.jece.2023.110293

[10] Yuan, K. *et al.* Facile synthesis and study of functional porous organic polyaminals with ultrahigh adsorption capacities and fast removal rate for rhodamine B dye. *Microporous Mesoporous Mater.* **344**, 112234 (2022). 10.1016/j.micromeso.2022.112234

[11] Dutta, D., Cole, N., Kumar, N. & Willcox, M. D. Broad spectrum antimicrobial activity of melimine covalently bound to contact lenses. *Invest. Ophthalmol. Visual Sci.* **54**(1), 175–182 (2013). 23211820 10.1167/iovs.12-10989

[12] Carmona-Ribeiro, A. M. & Araújo, P. M. Antimicrobial polymer− based assemblies: a review. *Int. J. Mol. Sci.* **22**(11), 5424 (2021). 34063877 10.3390/ijms22115424 PMC8196616

[13] Qiu, H. *et al.* The mechanisms and the applications of antibacterial polymers in surface modification on medical devices. *Front. Bioeng. Biotechnol.* **8**, 910 (2020). 33262975 10.3389/fbioe.2020.00910 PMC7686044

[14] Sun, C., Wang, X., Dai, J. & Ju, Y. Metal and metal oxide nanomaterials for fighting planktonic bacteria and biofilms: a review emphasizing on mechanistic aspects. *Int. J. Mol. Sci.* **23**(19), 11348 (2022). 36232647 10.3390/ijms231911348 PMC9569886

[15] Fan, D. *et al.* Functional insights to the development of bioactive material for combating bacterial infections. *Front. Bioeng. Biotechnol.* **11**, 1186637 (2023). 37152653 10.3389/fbioe.2023.1186637 PMC10160456

[16] McKeown, N. B. Polymers of intrinsic microporosity (PIMs). *Polymer* **202**, 122736 (2020). 10.1016/j.polymer.2020.122736

[17] Soria, R. B., & Luis, P. Antifouling membranes for polluted solvents treatment. In *Current Trends and Future Developments on (Bio-) Membranes* 2023,: 295–334. Elsevier.

[18] Yang, S. *et al.* Self-assembled short peptides: recent advances and strategies for potential pharmaceutical applications. *Mater. Today Bio.* **1**(20), 100644 (2023). 10.1016/j.mtbio.2023.100644 PMC10199221 37214549

[19] Antunes, J. C. *et al.* Recent trends in protective textiles against biological threats: a focus on biological warfare agents. *Polymers* **14**(8), 1599 (2022). 35458353 10.3390/polym14081599 PMC9026340

[20] Younis, S. A., Lim, D. K., Kim, K. H. & Deep, A. Metalloporphyrinic metal-organic frameworks: controlled synthesis for catalytic applications in environmental and biological media. *Adv. Colloid Interface Sci.* **2020**(277), 102108 (2020). 10.1016/j.cis.2020.102108 32028075

[21] (a)-Song, Y., Phipps, J., Zhu, C., & Ma, S. Porous materials for water purification. *Angewandte Chemie*, 2023, *135*(11). (b)- He, J., Feng, Y., Jiang, J. et al. Preparation and characterization of a sustained-release antibacterial melamine-impregnated paper based on Ag-BTC. *J Mater Sci* ,2023, 58, 6727–6742. 10.1007/s10853-023-08436-0 10.1002/anie.202216724 36538551

[22] (a)- Liao L. , Li M. , Yin Y. , Chen J. , Zhong Q. , Du R. , Liu S. , He Y. , Fu W. , Zeng, F. Advances in Synthesis of Covalent Triazine Framework, *ACS Omega,* 2023 *8*, 5, 4527–4542 10.1021/acsomega.2c06961 (b)- Liu, M., Guo, L., Jin, S., & Tan, B. (2019). Covalent triazine frameworks: synthesis and applications. Journal of materials chemistry A, 2019, 7(10): 5153–5172. 10.1021/acsomega.2c06961 PMC9909813 36777586

[23] Shifrina, Z. B., Matveeva, V. G. & Bronstein, L. M. Role of polymer structures in catalysis by transition metal and metal oxide nanoparticle composites. *Chem. Rev.* **120**(2), 1350–1396 (2019). 31181907 10.1021/acs.chemrev.9b00137

[24] Yang, C. *et al.* Nanofibrous porous organic polymers and their derivatives: from synthesis to applications. *Adv. Sci.* **11**(19), 2400626 (2024). 10.1002/advs.202400626 PMC11109660 38476058

[25] (a)- Abbott, S., & Holmes, N. *Nanocoatings: Principles and Practice: From Research to Production*. DEStech Publications, Inc., 2013.(b)- Goda, E.S Abu Elella M. H., Sohail M., Singu B. S., Pandit B., El Shafey A.M., Aboraia A. M. , Gamal H. , Hong S. E., Yoon K. R., N-methylene phosphonic acid chitosan/graphene sheets decorated with silver nanoparticles as green antimicrobial agents, *Int.J. Bio. Macromolecules*, 2021,182, 680–688 10.1016/j.ijbiomac.2021.04.024 33838196

[26] Yin, I. X. *et al.* The antibacterial mechanism of silver nanoparticles and its application in dentistry. *Int. J. Nanomed.* **15**, 2555–2562 (2020). 10.2147/IJN.S246764 PMC7174845 32368040

[27] Li, L. *et al.* Silver nanoparticles induce protective autophagy via Ca 2+ /CaMKKβ/AMPK/mTOR pathway in SH-SY5Y cells and rat brains. *Nanotoxicology* **13**(3), 369–391 (2019). 30729847 10.1080/17435390.2018.1550226

[28] Shanmuganathan, R. *et al.* An enhancement of antimicrobial efficacy of biogenic and ceftriaxone-conjugated silver nanoparticles: green approach. *Environ. Sci. Pollut. Res. Int.* **25**(11), 10362–10370 (2018). 28600792 10.1007/s11356-017-9367-9

[29] Hosnedlova, B., Kabanov, D., Kepinska, M. & Narayanan, B. Effect of biosynthesized silver nanoparticles on bacterial biofilm changes in s aureus and *E coli*.. *Nanomater. Basel* **12**(13), 2183 (2022). 10.3390/nano12132183 PMC9268453 35808019

[30] Saallah, S. & Lenggoro, I. W. Nanoparticles carrying biological molecules: recent advances and applications. *KONA Powder Part J.* **35**, 89–111 (2018). 10.14356/kona.2018015

[31] Fernando, S., Gunasekara, T. & Holton, J. Antimicrobial nanoparticles: applications and mechanisms of action. *Sri Lankan J. Infect. Dis.* **8**(1), 2–11 (2018). 10.4038/sljid.v8i1.8167

[32] (a)- Moritz M, Geszke-Moritz M. The newest achievements in synthesis, immobilization and practical applications of antibacterial nanoparticles. Chem Eng J. 2013, 228: 596–613 (b)- Goda E. S., Abu Elella, M.H., Hong, S.E. Pandit, B., Yoon K.R., Gamal H., Smart flame retardant coating containing carboxymethyl chitosan nanoparticles decorated graphene for obtaining multifunctional textiles. *Cellulose* 2021, 28, 5087–5105. 10.1007/s10570-021-03833-7 (c)- Abu Elella, M. H, Goda E. S, Yoon K.R., Hong, S. E. Morsy, M. S., Sadak R. A., Gamal H. , Novel vapor polymerization for integrating flame retardant textile with multifunctional properties, *Composites Comm.*, 2021, 24, ,100614, 10.1016/j.coco.2020.100614.

[33] Barroso-Solares, S., Cimavilla-Roman, P., Rodriguez-Perez, M. A. & Pinto, J. Non-invasive approaches for the evaluation of the functionalization of melamine foams with in-situ synthesized silver nanoparticles. *Polymers (Basel)* **12**(5), 996 (2020). 32344876 10.3390/polym12050996 PMC7285167

[34] Alkayal N.S.l, Alotaibi M.M., Tashkandi N.Y., Alrayyani M. A.,. Synthesis and characterization of bipyridine-based polyaminal network for CO_2_ capture. *Polym. Basel* **14**(18), 3746 (2022). 10.3390/polym14183746 PMC9502079 36145890

[35] Md, S., Abdullah, S., Awan, Z. A. & Alhakamy, N. A. Smart Oral pH-responsive dual layer nano-hydrogel for dissolution enhancement and targeted delivery of naringenin using protein-polysaccharides complexation against colorectal cancer. *J. Pharm. Sci.* **111**(11), 3155–3164 (2022). 36007557 10.1016/j.xphs.2022.08.019

[36] Balouiri, M., Sadiki, M. & Ibnsouda, S. K. Methods for in vitro evaluating antimicrobial activity: a review. *J. Pharm. Anal.* **6**(2), 71–79 (2016). 29403965 10.1016/j.jpha.2015.11.005 PMC5762448

[37] Yang, Z. *et al.* Breast cancer resistance protein (ABCG2) determines distribution of genistein phase II metabolites: reevaluation of the roles of ABCG2 in the disposition of genistein. *Drug Metab. Dispos.* **40**(10), 1883–1893 (2012). 22736306 10.1124/dmd.111.043901 PMC3463821

[38] Md, S. *et al.* Formulation design, statistical optimization, and in vitro evaluation of a naringenin nanoemulsion to enhance apoptotic activity in a549 lung cancer cells. *Pharmaceuticals* **13**(7), 152 (2020). 32679917 10.3390/ph13070152 PMC7407592

[39] Bahrami, A. Effect of curcumin and its derivates on gastric cancer: molecular mechanisms. *NuCancer* **73**(9), 1553–1569 (2021). 10.1080/01635581.2020.1808232 32814463

[40] Martocq, L. & Douglas, T. E. L. Amine-rich coatings to potentially promote cell adhesion, proliferation and differentiation, and reduce microbial colonization: strategies for generation and characterization. *Coatings* **11**(8), 983 (2021). 10.3390/coatings11080983

[41] Abdullah, S., El Hadad, S. & Aldahlawi, A. The development of a novel oral 5-Fluorouracil in-situ gelling nanosuspension to potentiate the anticancer activity against colorectal cancer cells. *Int. J. Pharm.* **613**, 121406 (2022). 34968681 10.1016/j.ijpharm.2021.121406

[42] Md, S. *et al.* Ambroxol hydrochloride loaded gastro-retentive nanosuspension gels potentiate anticancer activity in lung cancer (A549) cells. *Gels* **7**(4), 243 (2021). 34940303 10.3390/gels7040243 PMC8700943

